# Heterogeneous Supersaturation in Mixed Perovskites

**DOI:** 10.1002/advs.201903166

**Published:** 2020-02-08

**Authors:** Chih Shan Tan, Yi Hou, Makhsud I. Saidaminov, Andrew Proppe, Yu Sheng Huang, Yicheng Zhao, Mingyang Wei, Grant Walters, Ziyun Wang, Yongbiao Zhao, Petar Todorovic, Shana O. Kelley, Lih Juann Chen, Edward H. Sargent

**Affiliations:** ^1^ Department of Electrical and Computer Engineering University of Toronto 10 King's College Road Toronto Ontario M5S 3G4 Canada; ^2^ Frontier Research Center on Fundamental and Applied Sciences of Matters Department of Materials Science and Engineering National Tsing Hua University Hsinchu Taiwan 30043 Republic of China; ^3^ Department of Chemistry and Electrical and Computer Engineering Centre for Advanced Materials and Related Technologies (CAMTEC) University of Victoria 3800 Finnerty Rd Victoria BC V8P 5C2 Canada; ^4^ Department of Chemistry University of Toronto 80 St. George Street Toronto Ontario M5S 3G4 Canada; ^5^ Department of Pharmaceutical Sciences Leslie Dan Faculty of Pharmacy University of Toronto Toronto Ontario M5S 3M2 Canada

**Keywords:** defects, perovskites, traps, twins

## Abstract

Thin‐film solar cells based on hybrid lead halide perovskites have achieved certified power conversion efficiencies exceeding 24%, approaching those of crystalline silicon. This motivates deeper studies of the mechanisms that determine their performance. Twin defect sites have been proposed as a source of traps in perovskites, yet their origin and influence on photovoltaic performance remain unclear. It is found that twin defects—observed herein via both transmission electron microscopy and X‐ray diffraction—are correlated with the amount of antisolvent added to the perovskite and that twin defects in the highest‐performing perovskite photovoltaics are suppressed. Heterogeneous supersaturation nucleation is discussed as a contributor to efficient perovskite‐based optoelectronic devices.

## Graphical abstract

Twin defect sites are proposed as a source of traps in perovskites, yet their origin and influence on photovoltaic performance remain unclear. Here, it is found that twin defects are influenced by the antisolvent amount added to the perovskite, a finding seen both via transmission electron microscopy and X‐ray diffraction. Twin defects in the highest‐performing perovskites are suppressed.

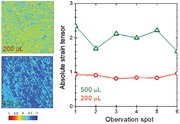

Perovskite solar cells have attracted interest in recent years and have achieved certified power conversion efficiencies (PCEs) of 23.3%.^1^, ^2^ Since early reports in 2009,^3^ one theme cutting across many reports has been the high defect tolerance of the perovskite material.^4^, ^5^, ^6^, ^7^, ^8^, ^9^ Continuing to advance the PCE of single‐junction perovskite solar cells toward the Shockley–Quessier limit^10^, ^11^, ^12^ will require the elimination of remaining defects that create trap states for charge carriers.^13^, ^14^

Defects have been observed to form in perovskite under nonideal processing conditions.^34^, ^35^, ^36^ Approaches to control defect densities in solution‐processed perovskite materials include the use of solvent engineering^15^, ^16^, ^17^, ^18^, ^19^, ^20^, ^21^, ^22^, ^23^, ^24^, ^25^, ^26^, ^27^, ^28^, ^29^, ^30^, ^31^, ^32^ to enlarge the grain size and reduce the density of defects at grain boundaries and the grain surface. Another approach uses antisolvents in a one‐step spin coating method to control heterogeneous nucleation via local supersaturation.^33^

Twin defects occur when two separate crystals symmetrically share a twin plane. Twin boundaries have been reported in metal oxides and associated with trap states near the conduction band in MgO and with a 300 meV splitting of interface states away from the conduction band in m‐HfO_2_.^37^ Prior experimental research has established the existence of twin defects inside perovskite crystals, yet the influence of twins on device properties has yet to be addressed.^35^, ^36^ Recently, density functional theory calculations have revealed that (111) twin defects may form in mixed‐ion perovskites. Twin defects induce the nucleation of Cs‐ and I‐rich secondary phases, leading to hole‐trapping defects near the valence band edge, thus forming trap states.^38^ This motivated us to explore further the possibility that twin defects could influence PCE and to strive to identify correlations between twin defects and PCE in perovskite solar cells.

Here we tune the twin defect density in metal halide perovskites using different amounts of antisolvent during the deposition of perovskite films. We use transmission electron microscopy (TEM) to observe twin defects and to quantify strain tensors induced by them, and we find that lower twin defect densities correlate with higher PCEs of solar cells. The (100) X‐ray diffraction (XRD) peak shows that (111) twin boundaries tend to build up stress on the (100) plane; the interplanar (100) spacing is largest when the twin defects are minimized. Further control over heterogeneous supersaturation nucleation may provide a path for further improvement of perovskite solar cells.

Scanning electron microscopy (SEM) images of perovskites treated with different amounts of chlorobenzene (CB) antisolvent (**Figure** **1**a–e; Figure S1, Supporting Information) show minimal variation in perovskite morphology. Atomic force microscopy (AFM) experiments show no strong dependence of roughness on the amount of antisolvent (Figure 1f; Figure S2, Supporting Information); the root mean square (RMS) roughness of perovskites films treated with different amounts of CB antisolvent ranges from 10.7 to 6.7 nm (Table S1, Supporting Information). We also found no appreciable difference in UV–vis spectra with the various antisolvent volumes (Figure 1g; Figure S3, Supporting Information).

**Figure 1:**
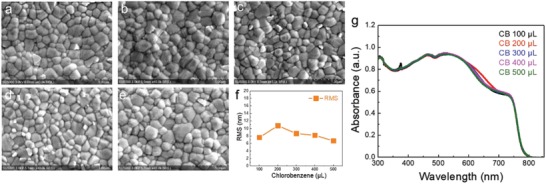
Scanning electron microscopy, film roughness, X‐ray diffraction, and UV–vis absorption spectrum of perovskites with different antisolvent treatments. SEM images of perovskites treated with a) 100, b) 200, c) 300, d) 400, and e) 500 µL of chlorobenzene antisolvent. f) RMS roughness. g) UV–vis absorption of perovskite thin films treated with different CB amounts.

The TEM analysis of perovskites shows variations in twin defect densities (**Figure** **2**). High‐resolution (HR) TEM images for the 200 and 500 µL CB antisolvent treatments are shown in Figure 2a,e, respectively. Each shows that the perovskite is of a cubic, *Pm* $\bar{3}$ *m* space group, and crystal lattice system. However, the 500 µL CB antisolvent perovskite exhibits more traces of (111) twin planes than that of the 200 µL, and the trace of (111) twin plane is readily discerned from the filtered (111) image for the 500 µL (Figure 2b,f). The filtered HRTEM images in Figure 2b,f only include the (111) scattered beams and exclude the other scattered beams.

**Figure 2:**
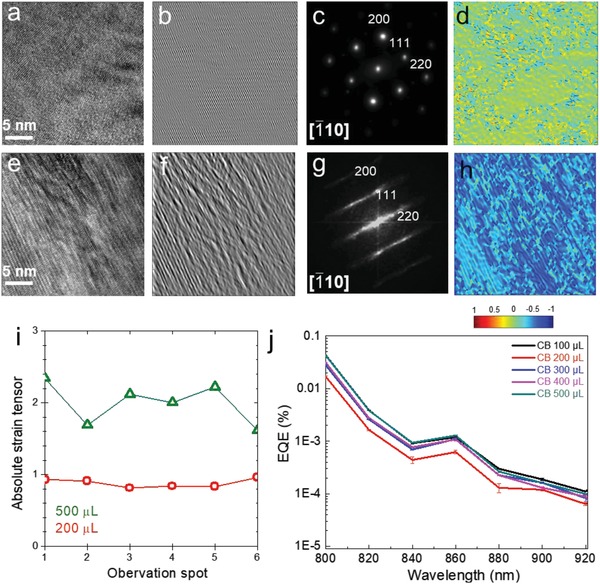
Twin defects and strain in perovskites treated with 200 and 500 µL of antisolvent. High‐resolution TEM images of perovskites treated with a) 200 and e) 500 µL of antisolvent. b,f) (111) filtered images of (a) and (e), respectively. c,g) FFT of (a) and (e), respectively. Perovskite films treated with 500 µL of antisolvent show twin defects while those treated with 200 µL do not. d,h) Normalized strain tensor (*ε_xy_*) distributions calculated from images (a) and (e), respectively. Color contour is showing the calculated values of the normalized strain tensor. i) The absolute value of the strain tensor (*ε_xy_*)_max_ took at different observation points for perovskite films treated with 200 and 500 µL of antisolvent. j) High‐dynamic‐range EQE. Error bars represent the standard deviation in the measurements of perovskite thin films treated with different antisolvent amounts.

Fast Fourier transforms (FFTs) of the high‐resolution images (Figure 2c,g) show that perovskites treated with 500 µL CB have more twin defects than those treated with 200 µL. To provide a quantitative estimation proportionate of the defect density, we used strain tensor analysis (*ε_xy_*_,_ see the details of calculation in the Experimental Section) in six areas of each sample (Figure 2d–h; Figures S4–S8, Supporting Information). We found the lowest and highest values of strain tensor for 200 and 500 µL of CB, respectively.

High‐resolution XRD patterns show that the position of the most prominent diffraction peak, (100), varies with the amount of antisolvent (**Figure** **3**a). The unfavorable treatments lead to shifts of the (100) peak toward higher angles. From Bragg's law, we further calculate the corresponding *d* spacings of the (100) peaks (Figure 3b). Such a peak shift suggests distortion inside perovskite. The interplanar (001) spacing is the largest in the absence of twin defects in samples treated with 200 µL antisolvent. Figure 3c depicts the possible tilting of the (100) plane by the (111) twin plane; this would produce a variation of the *d* spacing for the (100) plane. To exclude effects due to variations in elemental composition, we performed electron probe microanalyzer‐wavelength dispersive spectroscopy (EPMA‐WDS) and found that the compositions are the same for perovskites treated with different amounts of antisolvent (Tables S2 and S3, Supporting Information). We conclude that the observed difference in interplanar spacing is due to the twin defect densities within the perovskite grains.

**Figure 3:**
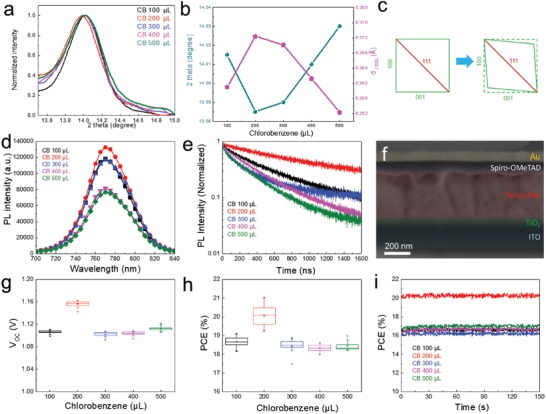
(100) X‐ray diffraction, photoluminescence, solar cell cross‐section, and photovoltaic characteristics. a) (100) XRD peak. b) Distortion of the (100) *d* spacing for perovskites treated with different amounts of antisolvent. c) Illustration the deformation of the (100) *d* spacing by the (111) twin plane. d) PL spectra. e) Time‐resolved PL. f) The cross‐section SEM image of the device. g) *V*_OC_, h) PCE, and i) steady‐state power output of photovoltaic devices with perovskites treated with different amounts of antisolvent.

To examine the properties of perovskites with different twin defect densities, we performed photoluminescence (PL) emission measurements and measured carrier lifetimes using time‐resolved PL (TRPL). PL measurements (Figure 3d) of perovskites prepared on glass using different amounts of antisolvent show that the PL peaks are all centered at 770 nm, but with different intensities. The TRPL traces (Figure 3e) and carrier lifetimes (Table S4, Supporting Information) change with varying amounts of antisolvent; longer carrier lifetimes are indicative of reduced recombination within the material, and therefore correspond to a higher quality material with lower trap state densities. A lifetime of 930 ns for the slower recombination (*t*_2_) was achieved with an antisolvent volume of 200 µL. The other films treated with 100, 300, 400, and 500 µL of antisolvent volume exhibited lifetimes of 520, 510, 410, and 330 ns, respectively. High‐dynamic‐range external quantum efficiency (EQE) measurements (measured in devices having the solar cell architecture shown in Figure 2j) were carried out to evaluate the influence of antisolvent treatment on the trap density within the perovskite. The samples with 200 µL antisolvent show a slightly decreased density of trap states. The PL lifetimes of perovskites are usually determined by all different types of defects density, such as vacancies, interstitials, antisites, and twin defects. However, both XRD *d*‐spacing variation and TEM images show dramatically increased twin defects in the perovskite thin films. Based on our current characterizations, we cannot differentiate the impacts of vacancies, interstitials, and antisites on the perovskite optoelectronic properties. Although we cannot totally rule out the influence of antisolvent volume on other types of defects, our TEM and XRD results led us to rationalize that the twin defects may have a strong influence on PL properties and *d*‐spacing variations.

To study the correlation of twin defect density with PCE, we fabricated solar cells in a planar architecture (ITO/TiO_2_/perovskite/Spiro‐OMeTAD/Au). Figure 3f shows a cross‐sectional SEM image of the solar cell. We found that the PCEs depend on the volume of antisolvent (Figure 3g–i; Figure S10, Supporting Information). A maximum PCE was achieved by using 200 µL of antisolvent, resulting in a stabilized power output (SPO) of 20%, while the other antisolvent amounts showed SPOs in the range of 16–17%. We point out that TEM data do not offer broad‐area analysis, and this limits the ability to draw conclusions regarding meaningful correlations with device performance and lifetime (inherently macroscopic parameters); however, our TEM images have the benefit of revealing crystal information at the nanoscale inside the perovskite film. For a better comparison of PCE and strain, XRD enables statistical analysis via (100) peak shifting. When we analyze strain variation inside the perovskite with different solvent volumes, including 100–500 µl, we find that the XRD shifts correlate with device PCE and PL lifetime results. Based on these TEM results, we did not observe any obvious variation of vacancies, interstitials, and antisites defects, but we do observe the different densities of twin defects, which correlate well with TRPL and PCE variation. Based on these experimental results, we believe that the twin defect density variation plays a bigger role in the TRPL and PCE than any other types of defects.

In summary, the influence of antisolvent on perovskite crystals is not evident in morphology, roughness, and absorption; whereas differences in twin defect densities are seen in TEM and strain tensor analysis as well as X‐ray diffraction. With an optimal amount of antisolvent, perovskite thin films show lower twin defect densities and correspondingly longer carrier lifetimes and superior device performance. This study provides evidence for a link between twin defects and perovskite solar cell performance and suggests that further reductions in defects within perovskite grains may contribute to further advancing multication perovskite solar cell performance.

## Experimental Section

### Perovskite Precursor Solution and Film Preparation

A 1.4 m precursor solution of Cs_0.05_FA_0.81_MA_0.14_PbI_2.55_Br_0.45_ was used to make the perovskite films. The precursors were dissolved in dimethyl sulfoxide (anhydrous, ≥99.9% (Sigma‐Aldrich)) and *N*,*N*‐dimethylformamide (anhydrous, 99.8% (Sigma‐Aldrich)) with proportions of 1:4. The precursor solution was heated at 60 °C to dissolve the precursor salts. The solution was then spin‐cast on substrates at 1000 rpm for 10 s and then at 6000 rpm for 40 s. CB (anhydrous, 99.8% (Sigma‐Aldrich)) antisolvent, of varying amount, was dropped on the film during the 6000 rpm spin step with 20 s of spin‐time remaining. Lastly, the perovskite films were annealed at 100 °C on a hot plate for 20 min.

### Photovoltaic Device Fabrication and Testing

Device fabrication followed methods reported in a previous paper.^39^ A 5 m TiO_2_ solution (anhydrous methanol: chloroform = 1:1) was spin‐cast onto ITO substrates using a 3000 rpm spin speed. The films were then annealed at 170 °C for 25 min using a hot plate. This produced films with ≈60 nm thickness. A 1.4 m precursor solution of Cs_0.05_FA_0.81_MA_0.14_PbI_2.55_Br_0.45_ was used to deposit perovskite films on top of the TiO_2_. The solution was spin‐cast at 1000 rpm spin for 10 s followed by spinning at 6000 rpm for 40 s. This formed ≈400 nm thick Cs_0.05_FA_0.81_MA_0.14_PbI_2.55_Br_0.45_ layers. The hole transport material (Spiro‐OMeTAD) was spin‐cast (4000 rpm 30 s) on top of the perovskite. The Spiro‐OMeTAD solution contained 0.1 g of Spiro‐OMeTAD, 37 µL of tert‐butylpridine, and 51 µL Li salt solution (bis(trifluoromethane)sulfonamide lithium salt 300 mg mL^−1^ in acetonitrile) dissolved in 1.5 mL of chlorobenzene. The hole transport layer, Spiro‐OMeTAD, was ≈100 nm thick. E‐beam evaporation of Au (100 nm) produced the top electrode. The final PV devices were composed of ITO (150 nm)/TiO_2_ (60 nm)/perovskite (≈400 nm)/Spiro (≈100 nm)/Au (100 nm). A Keithley 2400 source‐meter was used with a solar simulator (Newport, Class A) providing an irradiance of 100 mW cm^−2^ to measure current density–voltage (*J*–*V*) characteristics. The *J*–*V* scan rate was 50 mV s^−1^ with a delay time of 200 ms, and a voltage step of 10 mV. The active device area was 0.049 cm^2^.

### Perovskite Film Measurements

PL and PL time delay measurements were done using a Horiba Fluorolog time corrected single‐photon counting system with photomultiplier tube detectors with a 723 nm laser diode. XRD measurements were done using a D8 Discover X‐ray Diffraction System (Bruker) with a 0.154058 nm wavelength source (Copper Kα). TEM was conducted using a 300 kV Hitachi HF‐3300. Spin coating the perovskite film on the carbon support film (Ted Pella 01800‐F) with a concentration of 1.4 m would form 400 nm thick perovskite films, which are too thick for TEM examination. Thus, the Cs_0.05_FA_0.81_MA_0.14_PbI_2.55_Br_0.45_ precursor solution was diluted, maintaining the same ratio with the antisolvent, and deposited following the same procedure as the device films. An Asylum Research Cypher system was used for AFM. EPMA‐WDS was done using a JEOL JXA8230 5‐WDS system with perovskite films on ITO and glass substrates. To capture the cross‐sectional view of the photovoltaic device, focused ion beam processing was done using an FEI Helios Nano 600 Dual Beam system. The UV–vis absorption measurement was done using a Perkin Elmer system equipped with a 150 mm integrating sphere. EQE measurements were done using a Newport Quantx 300 system. The high‐dynamic‐range EQE measurement had a sensitivity set by the preamplifier to be 5 nA V^−1^. This ensured an appropriate resolution of the EQE measurement in the 800 to 980 nm region.

The strain tensor calculation from the diffraction pattern of transmission electron microscopy is described in the previous research.^40^, ^41^ The components of the strain tensor (*ɛ_xy_*) of the high‐resolution image at any position are obtained by differentiation of the small displacements

(1) $$\varepsilon_{xy}=\;\frac{1}{2}\;\left(\frac{\partial u_{x}}{\partial y}\;+\;\frac{\partial u_{y}}{\partial x}\right)$$

The Fourier filtered lattice pattern using (111) Bragg reflection of the high‐resolution twin defect perovskite region allows mapping the strain tensor.

## Conflict of Interest

The authors declare no conflict of interest.

## Supporting information

Supporting Information
