# Supplementary material for: Heterogeneous Supersaturation in Mixed Perovskites
Source: Adv Sci (Weinh). 2020 Feb 8;7(7):1903166. doi: 10.1002/advs.201903166 (PMC7140989; doi:10.1002/advs.201903166)
Supplement: Supplementary file 1 — Supporting Information [file ADVS-7-1903166-s001.pdf]

Copyright WILEY-VCH Verlag GmbH & Co. KGaA, 69469 Weinheim, Germany, 2020.

ADVANCED SCIENCE
Open Access

Supporting Information

for *Adv. Sci.,* DOI: 10.1002/advs.201903166

Heterogeneous Supersaturation in Mixed Perovskites

Chih Shan Tan, Yi Hou, Makhsud I. Saidaminov, Andrew Proppe, Yu Sheng Huang, Yicheng Zhao, Mingyang Wei, Grant Walters, Ziyun Wang, Yongbiao Zhao, Petar Todorovic, Shana O. Kelley, Lih Juann Chen, and Edward H. Sargent**

Supporting Information

Heterogeneous supersaturation in mixed perovskites

Chih Shan Tan, Yi Hou, Makhsud I. Saidaminov, Andrew Proppe, Yu Sheng Huang, Yicheng Zhao, Mingyang Wei, Grant Walters, Ziyun Wang, Yongbiao Zhao, Petar Todorovic, Shana O. Kelley, Lih Juann Chen, Edward H. Sargent

Dr. Chih Shan Tan, Dr. Yi Hou, Mingyang Wei, Andrew Proppe, Yicheng Zhao, Dr. Makhsud I. Saidaminov, Grant Walters, Dr. Ziyun Wang, Dr. Yongbiao Zhao, Petar Todorovic, Prof. Edward H. Sargent
Department of Electrical and Computer Engineering, University of Toronto, 10 King's College Road, Toronto, Ontario M5S 3G4, Canada
E-mail: ted.sargent@utoronto.ca

Dr. Chih Shan Tan, Yu Sheng Huang, Prof. Lih Juann Chen
Frontier Research Center on Fundamental and Applied Sciences of Matters, Department of Materials Science and Engineering, National Tsing Hua University, Hsinchu, Taiwan 30043, R.O.C.
E-mail: ljchen@mx.nthu.edu.tw

Dr. Makhsud I. Saidaminov
Department of Chemistry and Electrical & Computer Engineering, Centre for Advanced Materials and Related Technologies (CAMTEC), University of Victoria, 3800 Finnerty Rd, Victoria, BC V8P 5C2, Canada

Andrew Proppe, Prof. Shana O. Kelley
Department of Chemistry, University of Toronto, 80 St. George Street, Toronto, Ontario, Canada, M5S 3G4.

Prof. Shana O. Kelley
Department of Pharmaceutical Sciences, Leslie Dan Faculty of Pharmacy, University of Toronto, Toronto, Ontario, Canada, M5S 3M2

Time-resolved photoluminescence decay analysis
To analyze the antisolvent amount effect of perovskite, we performed time-resolved photoluminescence (TRPL) decay measurements (Figs S12b and S12c). The PL decays for perovskite films are on a glass substrate. The PL decay curves were fitted with a biexponential rate law:
$Y = A_1 \exp(-t/t_1) + A_2 \exp(-t/t_2) + y_0$ (1)
Where A_1, A_2, and $A_3$are the relative amplitudes and t_1 and t_2 are the lifetimes for the fast and slow recombination, respectively. The decay is attributed mainly to trap-assisted recombination at defects.[14]

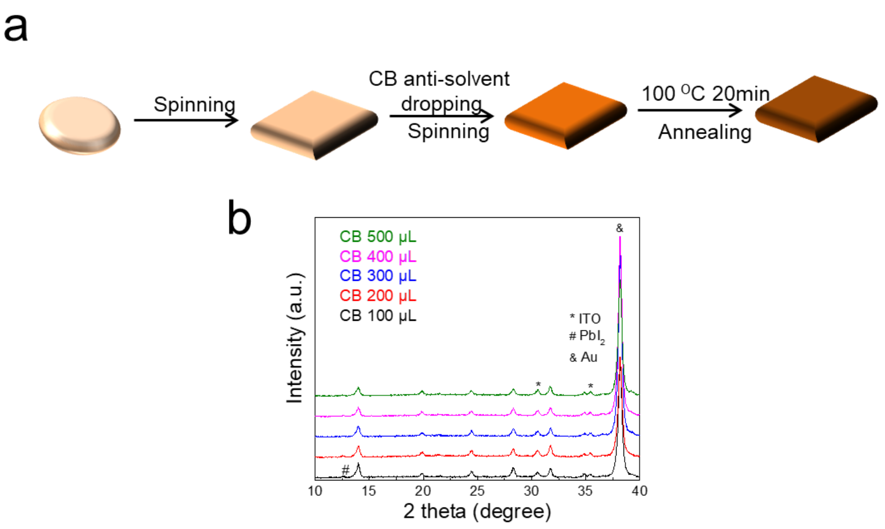

Figure. S1 a)The perovskite film formation processes and b) powder XRD with different antisolvent treatments.

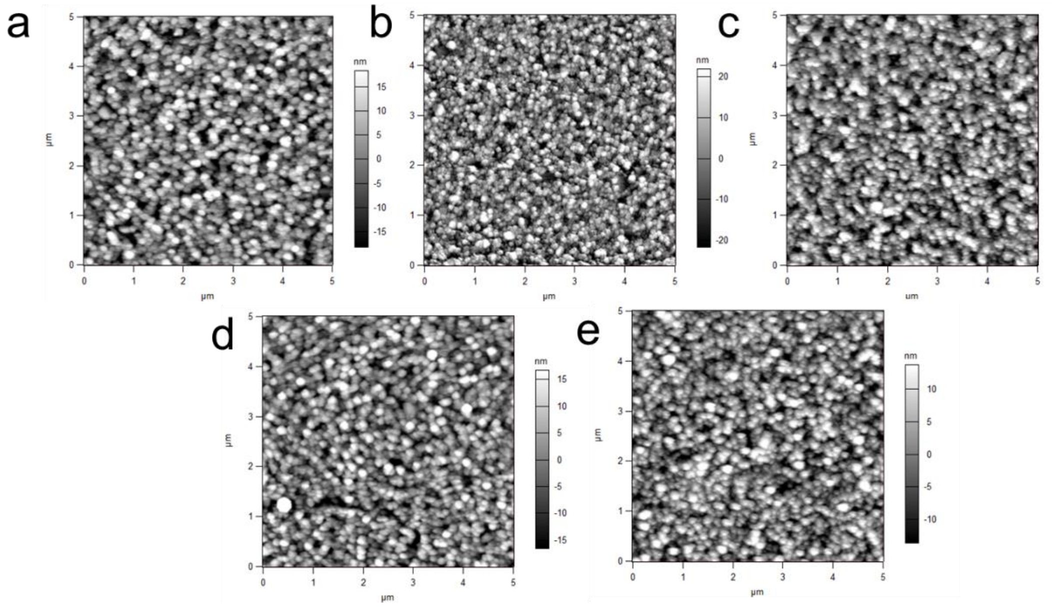

Figure. S2 AFM data of perovskite with different amounts antisolvent. a) AFM image with CB 100 μL. b) AFM image with CB 200 μL. c) AFM image with CB 300 μL. d) AFM image with CB 400 μL. e) AFM image with CB 500 μL.

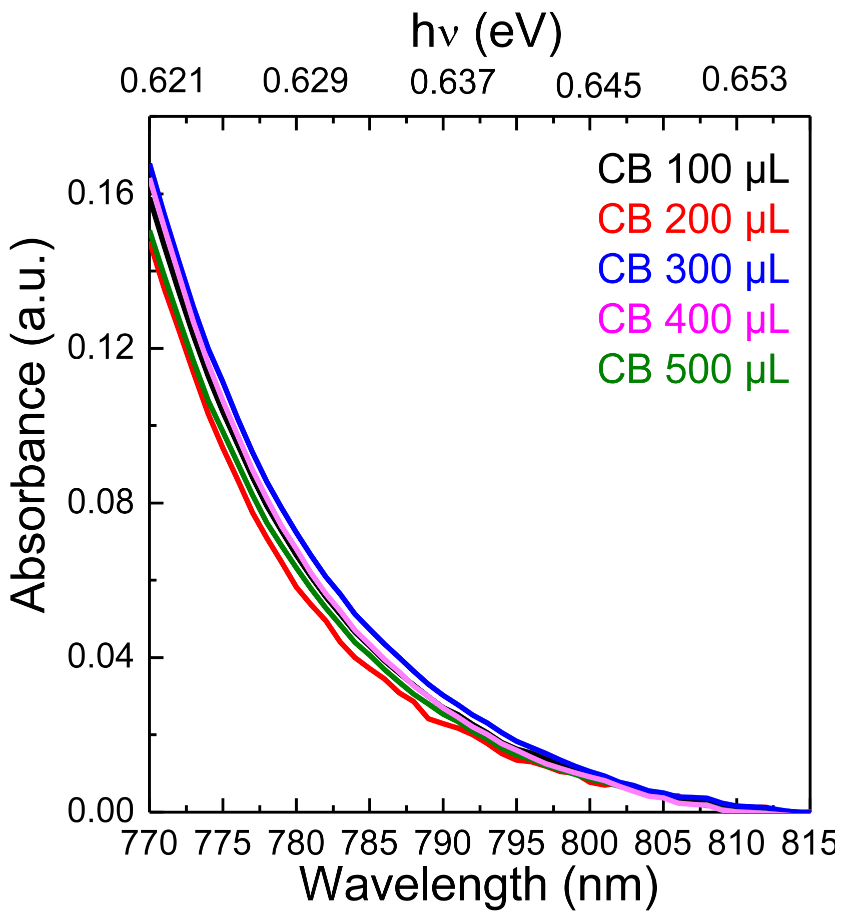

Figure S3. Low energy tail of the UV-visible absorption spectra for perovskite films with different amounts of antisolvent.

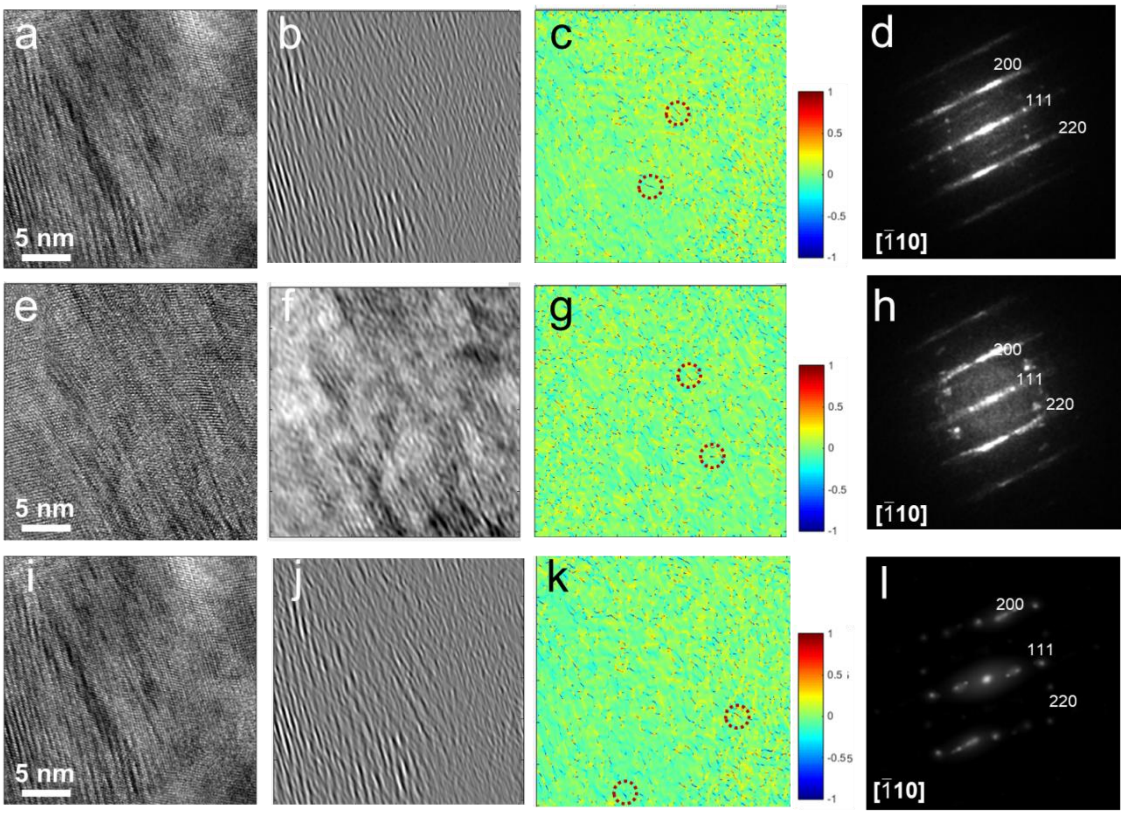

Figure S4. High-resolution TEM images (a, e, i), filtered twin defect images (b, f, j), normalized strain tensor (ε_{xy}) distribution (c, g, k), and Fast Fourier transform images (d, h, l) of CB 100 μL antisolvent treated perovskites. The red dashed circles are the twin defects of the highest strain tensor.

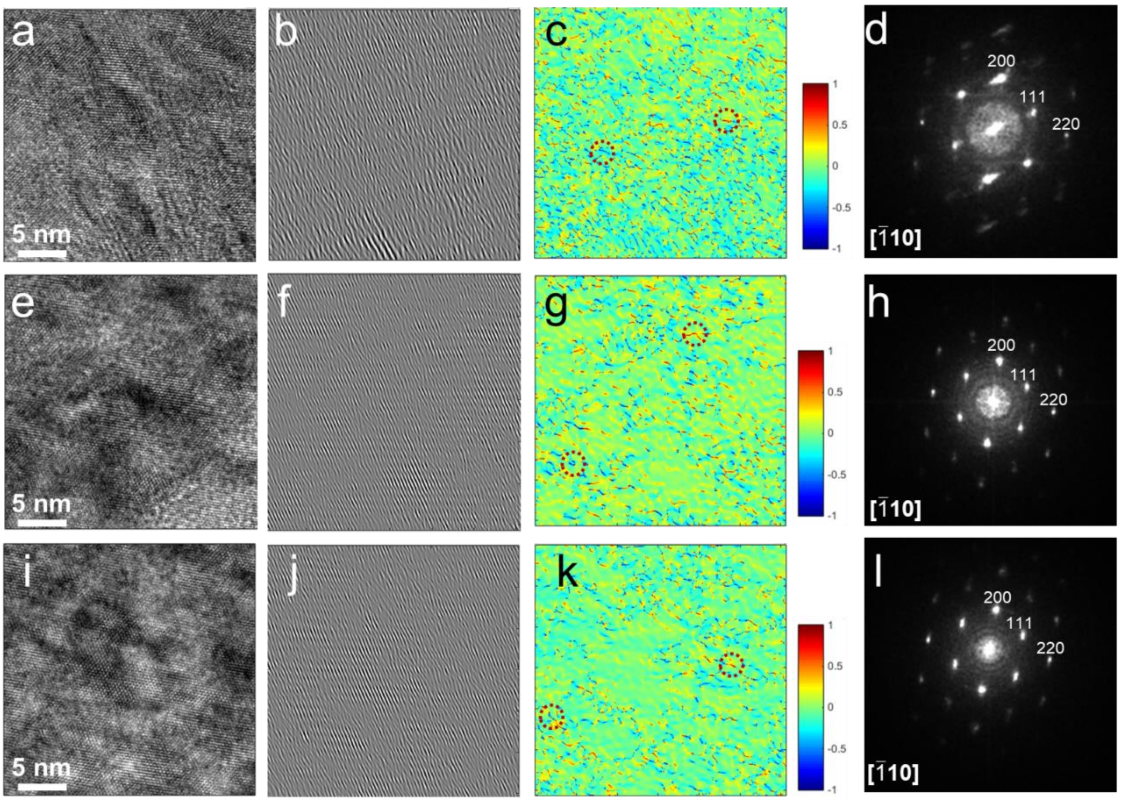

Figure S5. High-resolution TEM images (a, e, i), filtered twin defect images (b, f, j), normalized strain tensor (ε_{xy}) distribution (c, g, k), and Fast Fourier transform images (d, h, l) of CB 200 μL antisolvent treated perovskites. The red dashed circles are the twin defects of the highest strain tensor.

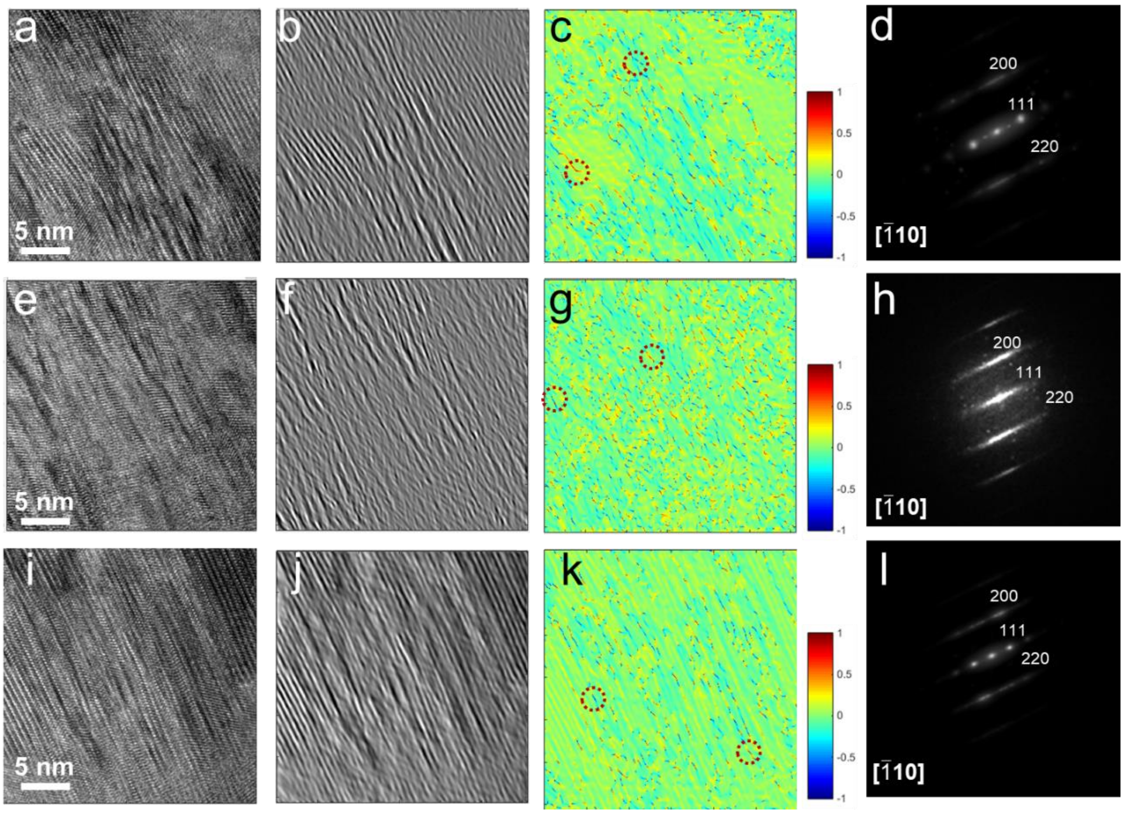

Figure S6. High-resolution TEM images (a, e, i), filtered twin defect images (b, f, j), normalized strain tensor (ε_{xy}) distribution (c, g, k), and Fast Fourier transform images (d, h, l) of CB 300 μL antisolvent treated perovskites. The red dashed circles are the twin defects of the highest strain tensor.

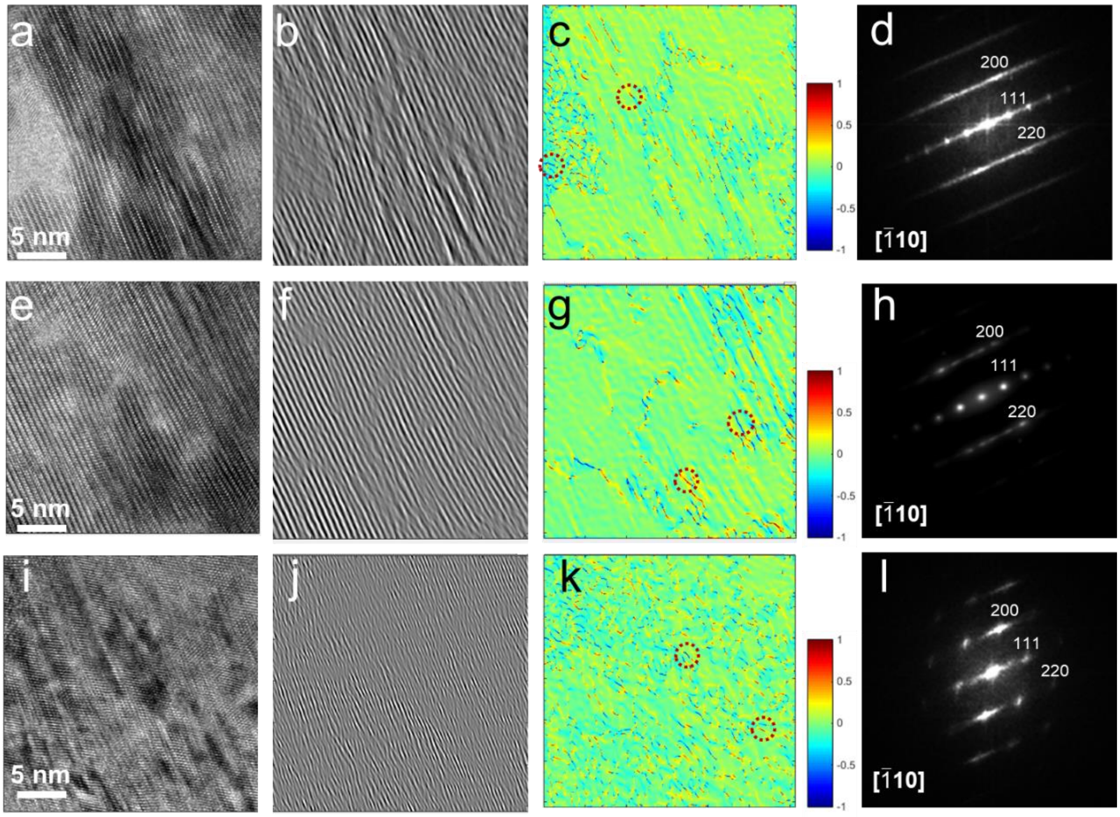

Figure S7. High-resolution TEM images (a, e, i), filtered twin defect images (b, f, j), normalized strain tensor (ε_{xy}) distribution (c, g, k), and Fast Fourier transform images (d, h, l) of CB 400 μL antisolvent treated perovskites. The red dashed circles are the twin defects of the highest strain tensor.

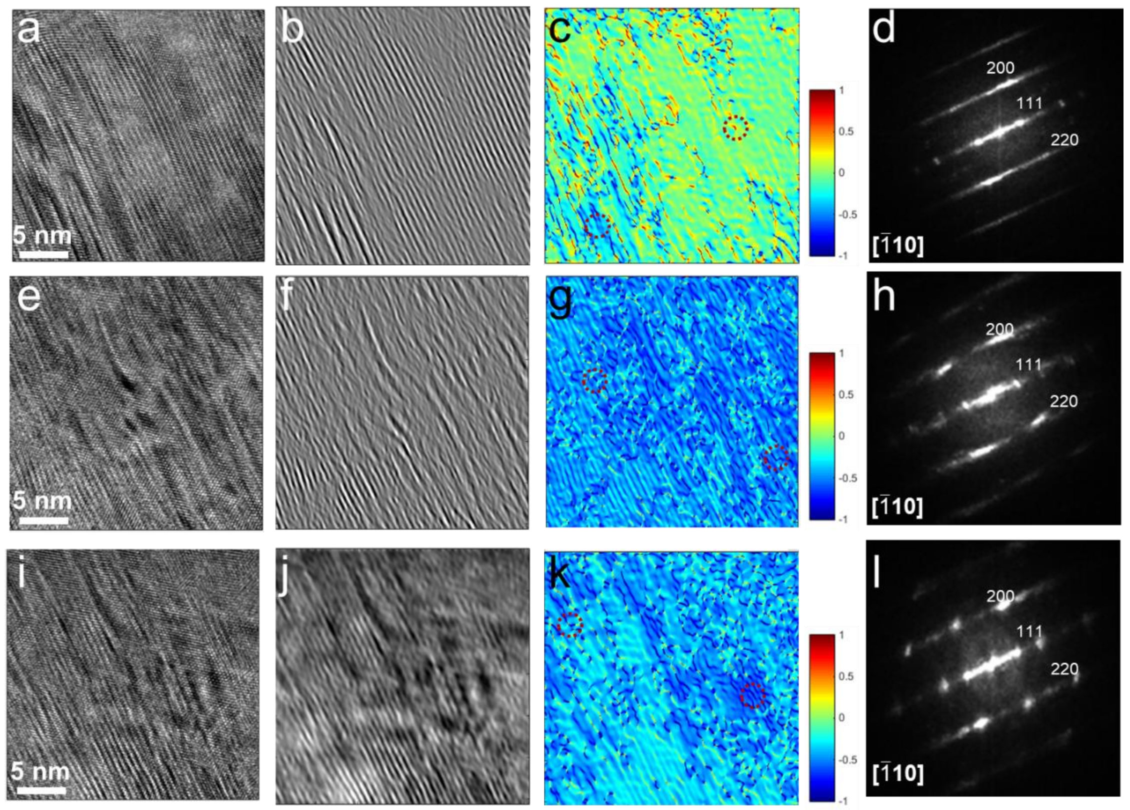

Figure S8. High-resolution TEM images (a, e, i), filtered twin defect images (b, f, j), normalized strain tensor (ε_{xy}) distribution (c, g, k), and Fast Fourier transform images (d, h, l) of CB 500 µL antisolvent treated perovskites. The red dashed circles are the twin defects of the highest strain tensor.

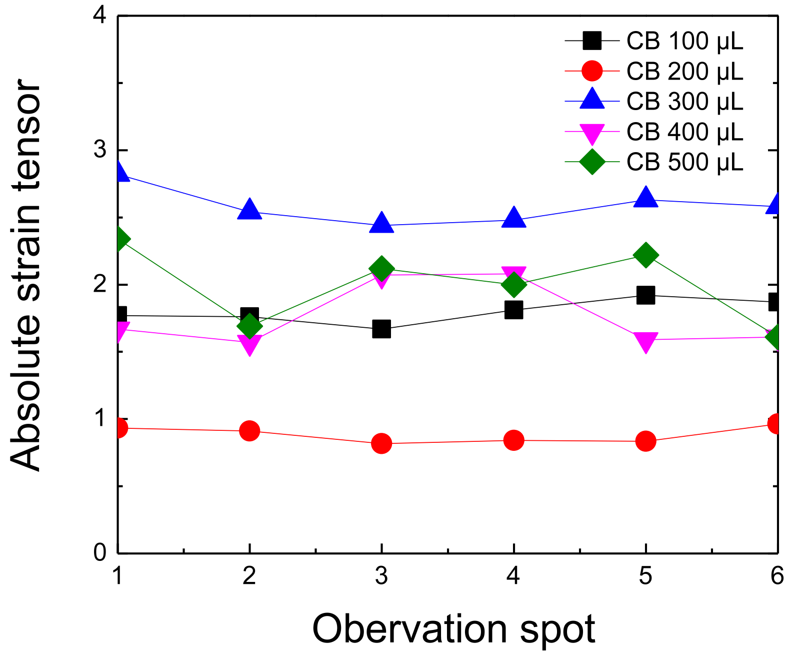

Figure S9. The absolute value of the strain tensor (εxy)max has taken at different observation points for perovskite films treated from 100 to 500 µL of antisolvent. The red circles in S4-S8 label the selection points.

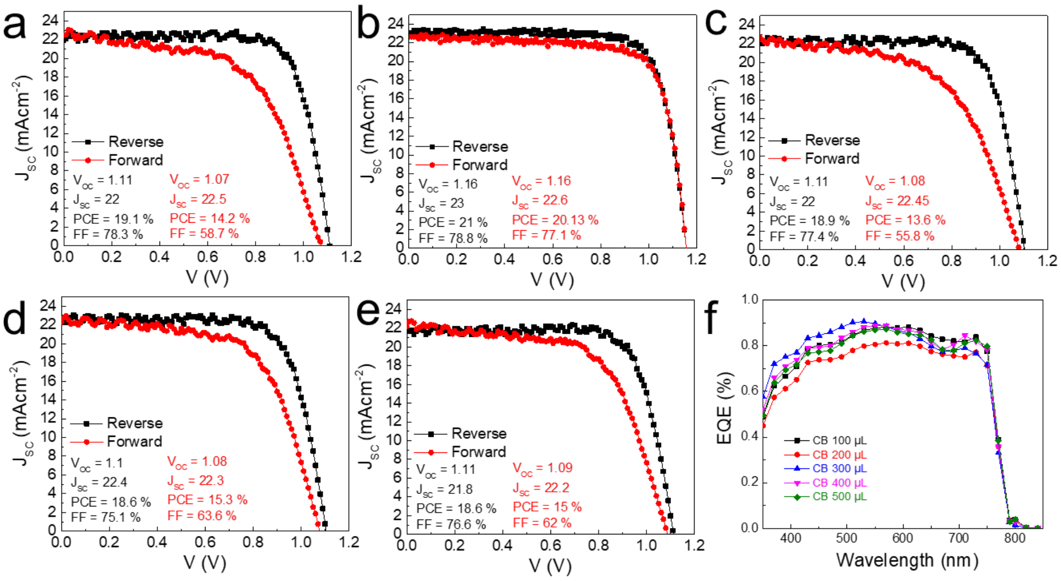

Figure. S10 The *J-V* curves, steady-state power output, and EQE of perovskite photovoltaics with different amount antisolvents. a) CB = 100 μL. b) CB = 200 μL.c) CB = 300 μL. d) CB = 400 μL. e) CB = 500 μL. e) EQE of different antisolvent.

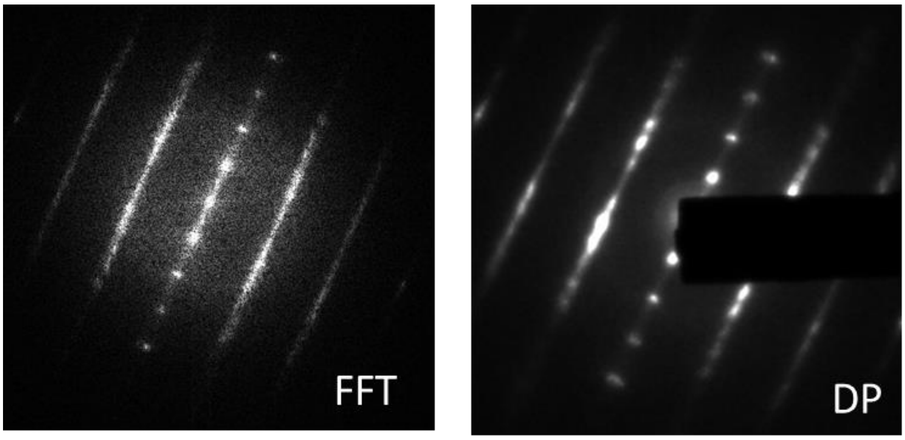

Figure. S11 The fast Fourier transform (FFT) and actually electron diffraction pattern (DP) of the same twin defect in perovskite area.

Table S1 The root mean square (RMS) of perovskite with different amounts of antisolvent.

	root mean square (RMS) (nm)
CB 100 μL	7.6
CB 200 μL	10.7
CB 300 μL	8.6
CB 400 μL	8.2
CB 500 μL	6.7

Table S2 The EPMA-WDS point analyses of the perovskite / ITO with different amounts of antisolvent treatment.

Point	Comment	Pb(Mass%)	Br(Mass%)	N(Mass%)	Cs(Mass%)	I(Mass%)	In(Mass%)	Sn(Mass%)	C(Mass%)	Total(Mass%)
1	CB 100 Mul-1	29.197	1.792	3.836	0.44	52.161	0	1.055	11.519	100
2	CB 100 Mul-2	29.735	1.788	3.646	0.396	51.915	0	1.213	11.307	100
3	CB 100 Mul-3	29.228	1.736	3.971	0.414	52.354	0	0.784	11.513	100
4	CB 200 Mul-1	31.291	1.896	3.205	0.557	55.267	3.834	1.27	2.68	100
5	CB 200 Mul-2	30.674	1.813	3.61	0.636	54.336	4.972	0.637	3.322	100
6	CB 200 Mul-3	29.9	1.898	3.425	0.554	55.364	4.887	1.328	2.644	100
7	CB 300 Mul-1	29.552	1.872	3.812	0.537	53.52	5.889	1	3.818	100
8	CB 300 Mul-2	29.548	1.728	3.731	0.569	53.682	5.66	1.101	3.981	100
9	CB 300 Mul-3	30.325	1.872	3.424	0.644	52.665	6.065	0.754	4.251	100
10	CB 400 Mul-1	30.723	1.924	3.528	0.446	54.783	4.767	0.769	3.06	100
11	CB 400 Mul-2	30.171	1.882	3.191	0.525	54.331	5.567	1.327	3.006	100
12	CB 400 Mul-3	29.415	1.696	3.386	0.464	52.213	6.516	1.035	5.275	100
13	CB 500 Mul-1	28.744	1.785	3.726	0.568	53.084	6.855	0.543	4.695	100
14	CB 500 Mul-2	29.183	1.675	3.189	0.589	52.16	6.89	0.696	5.618	100
15	CB 500 Mul-3	29.555	1.858	3.459	0.5	53.175	6.501	0.956	3.996	100

Point 1 to 3 are the spots on perovskite / ITO with CB 100 μL.
Point 4 to 6 are the spots on perovskite / ITO with CB 200 μL.
Point 7 to 9 are the spots on perovskite / ITO with CB 300 μL.
Point 10 to 12 are the spots on perovskite / ITO with CB 400 μL.
Point 13 to 15 are the spots on perovskite / ITO with CB 500 μL.

Table S3 The EPMA-WDS mapping summary of the averaged intensity of Pb, Br, N, Cs, I, and C elements on perovskite/glass in 600 x 427 µm area of perovskite with different amount antisolvent. The results showing the composition are nearly the same.

Averaged Intensity (counts per second / dwell time/pixel size/current)	Pb	Br	N	Cs	I	C
CB 100 ul	1.2	0.38	0.82	1.7	19.5	0.9
CB 200 ul	1.29	0.38	0.83	1.7	19.5	0.8
CB 300 ul	1.3	0.38	0.83	1.7	19.8	0.8
CB 400 ul	1.3	0.37	0.83	1.7	19.4	0.9
CB 500 ul	1.3	0.37	0.82	1.7	19.3	0.8
Mapping Condition						
Length	600	X	427	um		
Dwell Time	3	ms				
Accelerating voltage	12	kV				
Current	20	nA				
Pixel	600	X	427			
Pixel Size	1	X	1	um		

Table S4 *The PL lifetime, interplanar spacing values, strain tensor of perovskite films, and PCEs of solar cells.*

Sample	λ_{ems} (nm)	PCE(%)	A_1	t_2 (ns)	A_2	Angle (degree)	$d_{(100)}$ (Å)	Strain tensor
CB 100 µL	770	19.1	0.14	521	0.68	14.02	6.3614	1.8±0.2
CB 200 µL	770	21.0	0.10	932	0.69	13.99	6.3748	0.88±0.1
CB 300 µL	770	18.9	0.13	507	0.64	13.99	6.3726	2.58±0.2
CB 400 µL	770	18.6	0.15	408	0.71	14.01	6.3636	1.77±0.2
CB 500 µL	770	18.6	0.19	334	0.64	14.03	6.3548	1.99±0.2

Table S5 The EQE and J_{sc} from the *J-V* curve of perovskite with different amounts antisolvent.

CB (µL)	EQE J_{SC} (mA cm^{-2})	*J-V* curve J_{SC} (mA cm^{-2})
100	20.5	22.4
200	20.9	22.8
300	20.4	22.2
400	20.5	22.4
500	20.2	22
